# Infectious complications of targeted drugs and biotherapies in acute leukemia. Clinical practice guidelines by the European Conference on Infections in Leukemia (ECIL), a joint venture of the European Group for Blood and Marrow Transplantation (EBMT), the European Organization for Research and Treatment of Cancer (EORTC), the International Immunocompromised Host Society (ICHS) and the European Leukemia Net (ELN)

**DOI:** 10.1038/s41375-022-01556-7

**Published:** 2022-04-02

**Authors:** Georg Maschmeyer, Lars Bullinger, Carolina Garcia-Vidal, Raoul Herbrecht, Johan Maertens, Pierantonio Menna, Livio Pagano, Anne Thiebaut-Bertrand, Thierry Calandra

**Affiliations:** 1 Certified Cancer Center, Klinikum Ernst von Bergmann, Charlottenstrasse 72, D-14467 Potsdam, Germany; 2 Department of Hematology, Oncology and Tumor Immunology, Campus Virchow, Charité – Universitätsmedizin Berlin, German Cancer Consortium (DKTK), Partner Site Berlin, Berlin, Germany; 3 Infectious Diseases Department, Hospital Clinic-IDIBAPS, Villarroel 170, BCN 08036 Barcelona, Spain; 4 Department of Hematology, Institut de Cancérologie Strasbourg.Europe (ICANS) and Université de Strasbourg, Inserm, UMR-S1113/IRFAC, Strasbourg, France; 5 Department of Microbiology, Immunology, and Transplantation, KULeuven, Leuven and Department of Hematology, UZLEUVEN, Leuven, Belgium; 6 Department of Sciences and Technologies for Humans and the Environment, University Campus Bio-Medico of Rome, University Hospital Foundation Campus Bio-Medico of Rome, Rome, Italy; 7 Hematology Division, Dipartimento di Diagnostica per Immagini, Radioterapia Oncologica ed Ematologia, Fondazione Policlinico Universitario A. Gemelli – IRCCS and Università Cattolica del Sacro Cuore, Rome, Italy; 8 Department of Hematology, Grenoble University Hospital, Grenoble, France; 9 Infectious Diseases Service, Department of Medicine, Lausanne University Hospital, University of Lausanne, CH-1011 Lausanne, Switzerland

**Keywords:** Epidemiology, Acute myeloid leukaemia

## Abstract

The 9th web-based European Conference on Infections in Leukemia (ECIL-9), held September 16-17, 2021, reviewed the risk of infections and febrile neutropenia associated with more recently approved immunotherapeutic agents and molecular targeted drugs for the treatment of acute myeloid leukemia (AML) and acute lymphoblastic leukemia (ALL). Novel antibody based treatment approaches (inotuzumab ozogamicin, gemtuzumab ozogamicin, flotetuzumab), isocitrate dehydrogenases inhibitors (ivosidenib, enasidenib, olutasidenib), FLT3 kinase inhibitors (gilteritinib, midostaurin, quizartinib), a hedgehog inhibitor (glasdegib) as well as a BCL2 inhibitor (venetoclax) were reviewed with respect to their mode of action, their immunosuppressive potential, their current approval and the infectious complications and febrile neutropenia reported from clinical studies. Evidence-based recommendations for prevention and management of infectious complications and specific alerts regarding the potential for drug-drug interactions were developed and discussed in a plenary session with the panel of experts until consensus was reached. The set of recommendations was posted on the ECIL website for a month for comments from members of EBMT, EORTC, ICHS and ELN before final approval by the panelists. While a majority of these agents are not associated with a significantly increased risk when used as monotherapy, caution is required with combination therapy such as venetoclax plus hypomethylating agents, gemtuzumab ozogamicin plus cytotoxic drugs or midostaurin added to conventional AML chemotherapy.

## Introduction

For several decades, intensive combination chemotherapy, with or without hematopoietic cell transplantation, has been the backbone for the treatment of acute leukemia in younger and fit patients, whereas less toxic regimes (e.g., use of hypomethylating agents) have been used in older and unfit patients. However, over the past 10–15 years, tremendous progress has been made in deciphering the molecular pathogenesis and phenotypic diversity of acute leukemia. Several intracellular signaling pathways that are critical to the genesis of this disease have been identified. This new knowledge has also revealed various immunological and molecular therapeutic targets, paving the way for precision medicine approaches, both for acute myeloid leukemia and acute lymphoblastic leukemia. Recently, several small-molecule inhibitors and immunotherapies have been successfully introduced, either as single agents or in combination with standard-of-care therapies. The introduction or the addition of these novel therapies may potentially alter the landscape of infectious complications that are normally seen in these immuno-compromised leukemia patients. In addition, the potential for hazardous drug-drug interactions when combining these novel agents with currently used anti-infective drugs, either prophylactically or therapeutically, underscores the need for more individualized and personalized treatment. This position paper updates our knowledge of infections associated with these agents and provides recommendations for a rational clinical management of prevention and treatment of infections in leukemia patients treated with immunotherapeutic and molecular targeted antineoplastic agents, given with or without standard therapy.

## Methods

In early 2021, the Executive Committee of ECIL nominated a group of experts in clinical hematology and oncology, infectious diseases and clinical pharmacology. The panel of experts established a list of drugs and immune-based therapies to be reviewed. Subgroups of experts reviewed the literature on specific agents and agreed on timelines. A literature search (Medline) up to 1 September 2021, using the MeSH terms “(Substance name)” AND (1) “mode of action”, (2) “immunosuppression” OR “immune system” OR “immune defense” (3) “toxicity” OR “adverse” OR “infection” OR “fever” was set up and the state of drug approval in 2021 (globally and regionally) determined. Subgroup members selected relevant publications for data analysis and extracted all relevant data from clinical reports to prepare summaries on the mode of action, the impact on immune defense and the state of approval. They prepared sets of recommendations on diagnostic procedures in case of fever and/or infection, management strategies (including the need for antimicrobial prophylaxis) and handling of the drug. Panelists compiled a complete slide set discussed in several consecutive online group meetings and electronic communication until two weeks before the online ECIL-9 plenary meeting. Panelists received the consented slide set by email prior to the plenary. On the day of the meeting (September 17, 2021), the slides were presented by each subgroup of experts and interactively discussed during a 3-h session. Subgroup members reviewed in closed sessions the comments made by panel members and revised recommendations accordingly. The revised set of recommendations was presented to the ECIL-9 plenary in a late afternoon session on the same day and discussed until reaching consensus. The approved slide set was published on the ECIL website (https://ecil-leukaemia.com/en/resources/resources-ecil) for comments over a month (November 2021). Members of the expert subgroups approved the final set of recommendations. For grading of the strength of recommendation and the level of evidence, the European Society of Clinical Microbiology and Infectious Disease (ESCMID) grading system [1] was used (Table 1).

**Table 1:** Grading system used for strength of recommendation and quality of evidence (after (1)).

Strength of recommendation	
Grade	Definition
A	ECIL strongly supports a recommendation for use
B	ECIL moderately supports a recommendation for use
C	ECIL marginally supports a recommendation for use
D	ECIL supports a recommendation against use
Quality of evidence	
Level	Definition
I	Evidence from at least 1 properly designed randomized, controlled trial (orientated on the primary endpoint of the trial)
II*	Evidence from at least 1 well-designed clinical trial (including secondary endpoints), without randomization; from cohort or case-controlled analytic studies (preferably from > 1 center); from multiple time series; or from dramatic results of uncontrolled experiments
III	Evidence from opinions of respected authorities, based on clinical experience, descriptive case studies, or reports of expert committees
Added index for source of level ii evidence	
*Index	Source
r	Meta-analysis or systematic review of RCT
t	Transferred evidence, that is, results from different patient cohorts, or similar immune-status situation
h	Comparator group: historical control
u	Uncontrolled trials
a	Published abstract presented at an international symposium or meeting

## Results and recommendations

For a summary of drug characteristics, reported infectious complications and ECIL clinical practice recommendations see Table 2.

**Table 2:** Summary of drug characteristics, reported infectious complications and ECIL clinical practice recommendations for targeted drugs and biotherapies in acute leukemia.

Class of agents	Agent	Impact on immune system	Infectious events	ECIL recommendations
Anti-CD22 antibody-drug conjugate	Inotuzumab ozogamicin	No documented mechanism of immunosuppression	When combined with chemotherapy: febrile neutropenia, sepsis, pneumonia	• No specific antimicrobial prophylaxis (A-IIr) • No specific recommendation with the use of this drug in case of infection or fever • Special attention when combining this drug with other agents prolonging QT interval, such as levofloxacin or posaconazole (A-IIr)
Anti-CD33 antibody-drug conjugate	Gemtuzumab ozogamicin	No specific impact on immune defense except neutropenia	When given in combination with chemotherapy: febrile neutropenia, pneumonia, sepsis, fungal infection)	Recommended diagnostic procedures: • Standard of care in AML and neutropenic fever and/or infections (A-IIr) Treatment recommendations: • Standard of care in neutropenic fever and/or infections (A-IIr) Recommendations for prophylaxis: • Standard of care in AML, when given in combination (A-IIr) or high-dose GO for relapse (A-IIr) • No systemic antimicrobial prophylaxis when given as monotherapy (A-IIr) Recommendations on how to handle the drug in case of infection or fever: • Most infections occur subsequent to GO application, therefore no recommendation General recommendation • Careful monitoring of hepatotoxicity (A-I)
CD123 x CD3 bispecific dual-affinity retargeting antibody (DART)	Flotetuzumab	No documented mechanism of immunosuppression	No specific risks of infection in patients on flotetuzumab monotherapy	Recommended diagnostic procedures: • Standard of care in AML and neutropenic fever and/or infections (A-IIr) Treatment recommendations: • Standard of care in neutropenic fever and/or infections (A-IIr) Recommendations for prophylaxis: • No specific recommendation due to lack of data when given as monotherapy Recommendations on how to handle the drug in case of infection: • No specific recommendation due to lack of data when given as monotherapy
Isocitrate dehydrogenase (IDH)-1 and -2 inhibitors	Enasidenib, ivosidenib, olutasidenib	No documented mechanism of immunosuppression	Reports on severe differentiation syndrome which may mimic an infection (fever, acute respiratory distress, pulmonary infiltrates, pleural or pericardial effusion, hyperleukocytosis, renal impairment, multiorgan failure)	Recommended diagnostic procedures: • Standard of care in AML and neutropenic fever and/or infections (A-IIr) Treatment recommendations: • Standard of care in neutropenic fever and/or infections (A-IIr) Recommendations for prophylaxis: • No systemic antimicrobial prophylaxis when given as monotherapy (A-IIr) Recommendations on how to handle the drug in case of infection: • No specific recommendations
FLT3-Tyrosine Kinase Inhibitor, also active against receptor tyrosine kinases KIT and AXL	Gilteritinib	No documented mechanism of immunosuppression	Infections in relapsed and/or refractory AML patients on gilteritinib: febrile neutropenia, sepsis pneumonia	Recommended diagnostic procedures: • Standard of care in AML and neutropenic fever and/or infections (A-IIr) Treatment recommendations: • Standard of care in neutropenic fever and/or infections (A-IIr) Recommendations for prophylaxis: • No systemic antimicrobial prophylaxis when given as monotherapy (A-IIr) Recommendations on how to handle the drug in case of infection: • No specific recommendations Recommendations on how to handle the drug in case of concomitant administration of strong CYP3A4 inhibitors (e.g., itraconazole, posaconazole, voriconazole): • Close monitoring for QT interval prolongation (A-I) Recommendations on how to handle the drug if current medication includes a strong CYP3A4 inducer: • Avoid combination
Multi-tyrosine kinase inhibitor of Fms-Like Receptor Tyrosine Kinase 3 (FLT3), KIT, platelet derived growth factor receptor (PDGFR), vascular endothelial growth factor receptor 2 (VEGFR2) and members of the serine/threonine kinases of the protein kinase C (PKC) family	Midostaurin	No specific impact on immune defense except neutropenia	Febrile neutropenia, respiratory tract infections, sepsis, herpes simplex virus infections	Recommended diagnostic procedures in case of fever: • Standard of care for fever during cytarabine/anthracycline chemotherapy plus midostaurin therapy (A-IIr) Treatment recommendations: • Use of empirical antibiotic therapy during cytarabine/anthracycline chemotherapy plus midostaurin therapy in neutropenic fever and/or infections (A-IIr) Recommendations for antimicrobial prophylaxis: • Standard of care when midostaurin is given in combination with cytarabine/anthracycline chemotherapy (A-IIr) • No need for antibacterial or antifungal prophylaxis when given as monotherapy (A-IIr) Recommendations for concurrent use of CYP3A4 inducers or inhibitors: • Warning for drug-drug interaction (see package insert): strong CYP3A4 inhibitors (clarithromycin, antifungal triazoles, cobicistat, antiviral protease, integrase, NS5A or polymerase inhibitors used for HIV and/or hepatitis C treatment) may increase exposure to midostaurin or its metabolites. Consider alternative therapies that do not strongly inhibit CYP3A4 or monitor for increased risk of adverse reactions. • Avoid concomitant use of strong CYP3A4 inducers such as rifampin.
Selective Fms-Like Receptor Tyrosine Kinase 3 (FLT3) tyrosine kinase inhibitor	Quizartinib	No specific impact on immune defense apart from neutropenia.	When used in combination with chemotherapy: febrile neutropenia and bacterial infections (sepsis, urinary tract infection, respiratory tract infection)	Recommended diagnostic procedures: • Standard of care in AML and neutropenic fever and/or infections (A-IIr) Treatment recommendations: • Use of empirical antibiotic approach in neutropenic fever and/or infections (A-IIr) Recommendations for antibiotic prophylaxis: • Standard of care in AML, when given in combination (A-IIr) Recommendations for combination with CYP3A4 inducing or inhibiting agents: • See recommendations for gilteritinib
Hedgehog pathway inhibitor	Glasdegib	No specific impact on immune defense apart from neutropenia	When given in combination with chemotherapy: febrile neutropenia, pneumonia, sepsis	Recommended diagnostic procedures: • Standard of care in AML and neutropenic fever and/or infections (A-IIr) Treatment recommendations: • Standard of care in neutropenic fever and/or infections (A-IIr) Recommendations for prophylaxis: • Standard of care in AML, when given in combination with chemotherapy (A-IIr) Recommendations on how to handle the drug in case of infection: • No specific recommendations Recommendations on combination with CYP3A4 inhibiting or inducing agents: • Critical reconsideration of concomitant erythromycin, clarithromycin, ciprofloxacin, itraconazole, posaconazole, ketoconazole or voriconazole • Avoidance of combination with strong CYP3A4 inducers (see above)
B Cell Lymphoma (BCL)-2 protein inhibitor	Venetoclax	Neutropenia	When given in combination with 5-azacitidine: febrile neutropenia, pneumonia, sepsis	Recommended diagnostic procedures: • Standard of care in AML and neutropenic fever and/or infections (A-IIr) Treatment recommendations: • Standard of care in neutropenic fever and/or infections (A-IIr) Recommendations for prophylaxis: • Standard of care as for AML treatment with intensive chemotherapy (A-IIr) Further recommendations for prophylaxis: • Consider antibacterial and antifungal prophylaxis when hypomethylating agents are combined with venetoclax (A-IIr) Recommendations on how to handle the drug in case of infection: • Consider dose interruptions to allow for hematologic recovery in patients with a response (based on early bone marrow assessment, most importantly after the completion of cycle 1) (A-I) • Promote appropriate interruptions in venetoclax between treatment cycles to augment hematologic recovery (A-I) • In patients with good response but severe neutropenia, consider venetoclax dose reduction in subsequent courses (A-IIr) • If dose reduction is ineffective or not advised, consider prophylactic granulocyte colony-stimulating factor during remission for subsequent courses (C-IIt) Recommendations for concurrent use of venetoclax with antimicrobial agents: • Ensure proper venetoclax dose adjustments when venetoclax is combined with antibacterials such as ciprofloxacin or macrolides (A-I) • If posaconazole is given in combination with venetoclax, reduce venetoclax dose by 75% (A-I)

### Inotuzumab ozogamicin

Inotuzumab ozogamicin (InO) is used as targeted therapy for adults with relapsed and/or refractory CD22-positive B-cell precursor ALL. It binds with high affinity to CD22, a cell-surface antigen expressed by >90% of B-cell blasts in nearly all patients with B-cell ALL. InO is an IG4 antibody-drug conjugate composed of a humanized anti-CD22 monoclonal antibody conjugated to a derivative of the cytotoxic agent calicheamicin. The cytotoxic agent is released intracellularly and induces DNA strand cleavage, with the subsequent cell death mediated through calicheamicin-induced apoptosis and not by CD22 signaling. It was approved in 2017 for adults with relapsed or refractory B-cell precursor acute lymphoblastic leukemia. The impact of CD22 inhibition on immunity is unclear. CD22 regulates multiple B cell functions, but the role of CD22 in protection against pathogens is not well established.

In a phase 3 randomized clinical trial (RCT) in patients with relapsed or refractory ALL, comparing 109 InO treated patients with 109 patients receiving standard therapy, febrile neutropenia was noted in 11 vs 18% of patients, pneumonia in 4% vs 1%, sepsis 2% vs 5% and septic shock 1% vs 1% of patients respectively [2].

In a phase 2 RCT, InO in combination with low-intensity chemotherapy (mini-hyper-CVD) vs InO monotherapy in 59 vs 84 patients with relapsed or refractory B-precursor ALL, infections were noted in 73% vs 17% of patients [3].

In a phase 2 RCT, mini-hyper-CVD in combination with InO plus or minus blinatumomab maintenance was given as first salvage treatment to patients with relapsed/refractory B-precursor ALL; infections occurred in 67% of patients undergoing InO plus mini-hyper-CVD therapy, one patient had sepsis [4].

In a phase 3 RCT, InO vs standard therapy was administered to patients with relapsed/refractory B-precursor ALL; febrile neutropenia was noted in 11.6% vs 18.9%, sepsis in 2.4% vs 7%, septic shock in 1.8% vs 2.1%, pneumonia in 6.1% vs 0% and fungal infection in 0 vs 2.1% of patients [5].

Prolongation of QT interval has been observed in patients receiving InO. Therefore, the concomitant use of InO with medicinal products known to prolong the QT interval or induce Torsades de pointes must be carefully evaluated. The QT interval must be monitored in case of combination of these medicines [6].

Risk of infection associated with inotuzumab ozogamicin:

- Inotuzumab ozogamicin monotherapy does not increase the risk of infection.

Recommendations:

- No specific antimicrobial prophylaxis is needed (A-IIr).
- No specific recommendation with the use of this drug in case of infection or fever.
- Special attention when combining this drug with other agents prolonging QT interval, such as levofloxacin or posaconazole (A-IIr).

### Gemtuzumab ozogamicin

Gemtuzumab ozogamicin (GO) is a recombinant humanized anti-CD33 monoclonal antibody conjugated to a derivative of calicheamicin. This conjugated antibody is rapidly internalized and causes subsequent apoptosis. CD33 is expressed on monocytes, granulocytes, mast cells and myeloid progenitors. Apart from profound neutropenia, no specific impact on immune defense has been described for GO. It is approved for the treatment of AML expressing CD33 in patients. It is used in combination with daunorubicin and cytarabine, but may also be applied as a monotherapy.

In a randomized phase 3 trial in elderly, newly diagnosed AML patients (*n* = 237) unfit for induction chemotherapy, GO (6 mg/m^2^ day 1 plus 3 mg/m^2^ day 8) was compared with best supportive care (BSC) [7]. Infections (with no further details reported) were documented in 44.1% vs 42.1%, and febrile neutropenia in 35.1% and 34.2% of patients, respectively. There was no difference in grade ≥3 infections or FN.

In another randomized phase 3 trial in 472 newly diagnosed elderly AML patients [8], induction chemotherapy using mitoxantrone, cytarabine and etoposide with vs without preceding two GO doses of 6 mg/m^2^ on days 1 and 15 was administered. Infections (without further details reported) were documented in 55% of patients in both arms, and FN in 35% vs 31% of patients with vs without GO pretreatment.

GO in a dosage of 6 mg/m^2^ every 4 weeks was compared with no further treatment for consolidation in 232 AML patients ≥60 years of age in complete remission after two conventional chemotherapy induction cycles with daunorubicin plus cytarabine followed by high-dose cytarabine [9]. Infections grade 2–4 (no further details reported) were observed in 43% of patients undergoing up to three GO consolidation cycles, including sepsis in 10%. A fatal infection did not occur.

Standard remission induction chemotherapy (cytarabine plus daunorubicin, “7 + 3”) with or without GO (3 mg/m^2^ on days 1, 4 and 7) was given to 268 AML patients in a randomized phase 3 trial [10]. Infections were documented in 77.6% of patients with no difference between the two groups. Breaking down the types of infections and FN, no significant differences between the two patient groups were reported in the supplement to this publication.

Another randomized phase 3 trial in 586 AML patients [11] compared similar two treatment modalities with GO given in a dosage of 6 mg/m^2^ on day 4 of induction. Fatal infections were noted in 5 of 292 (1.7%) patients with GO and 2 of 294 (0.7%) of patients without GO. No further details were reported.

Induction chemotherapy with idarubicin, cytarabine, etoposide and all-trans retinoic acid with or without GO (3 mg/m^2^ day 1) was given to 549 patients with de novo, *nucleophosmin-1* (*NPM1*) mutated AML in a randomized phase 3 study [12]. All-grade infections emerged in 79% of 274 patients treated with additional GO vs 73% of 275 patients without GO. No further details were reported with respect to infections.

De novo AML patients (*n* = 46) were treated in a single-arm trial with induction chemotherapy including fludarabine, cytarabine and idarubicin plus GO (3 mg/m^2^ day 4), and the outcome was compared to a matched historical control group treated with the same induction chemotherapy without GO [13] Infections were noted in 16 of 46 (34.8%) patients treated with GO and 25 of 47 (53.2%) patients treated with chemotherapy alone. Details on infectious complications were not reported.

Patients with first AML relapse (*n* = 277) were treated with GO monotherapy in a dosage of 9 mg/m^2^ for two doses separated by two weeks in an open label phase 2 trial [14]. Grade 3 or 4 adverse events included sepsis (17%), fever (13%), pneumonia (8%) and FN (6%). Within 28 days after the last GO dose, 44 patients (16%) had died, 13 of them (4.7% of total) from infection.

In 48 patients with relapsed (first or higher relapse) or refractory AML salvage treatment with fludarabine, cytarabine, idarubicin and granulocyte colony-stimulating factor plus GO (9 mg/m^2^ day 8) was given and outcomes compared with 23 matched control patients treated with the same salvage regimen without GO [15]. Documented fungal infections were noted in 2 of 23 (9%) patients without GO and 9 of 48 (19%) patients with GO, the difference being statistically non-significant. The fungal infections were predominantly attributed to *Candida* species.

Severe hepatic adverse events may emerge in patients undergoing GO therapy. In vitro, N-acetyl gamma calicheamicin dimethyl hydrazide is primarily metabolized via nonenzymatic reduction. Therefore, coadministration of GO with inhibitors or inducers of cytochrome P450 or uridine diphosphate glucuronosyltransferase (UGT) drug metabolizing enzymes are unlikely to alter exposure to N-acetyl gamma calicheamicin dimethyl hydrazide.

Risk of infection associated with gemtuzumab ozogamicin:

- No specific risk of infection is associated with GO monotherapy.
- In large comparative trials on chemotherapy ± GO, no significant increase of neutropenic fever/infections was specifically attributable to GO.
- Types of infectious events reported were fever of unknown origin (FUO), pneumonia, sepsis and fungal infection, with no potentially GO-related infections.

Recommended diagnostic procedures:

- Standard of care in AML and neutropenic fever and/or infections (A-IIr).

Treatment recommendations:

- Standard of care in neutropenic fever and/or infections (A-IIr).

Recommendations for prophylaxis:

- Standard of care in AML, when given in combination (A-IIr) or high-dose GO for relapse (A-IIr).

- No systemic antimicrobial prophylaxis when given as monotherapy (A-IIr).

Recommendations on how to handle the drug in case of infection:

- Most infections occur subsequent to GO application, therefore no recommendation.

General recommendation:

- Careful monitoring of hepatotoxicity (A-I).

### Flotetuzumab

Flotetuzumab is an investigational CD123 x CD3 bispecific dual-affinity retargeting antibody (DART) molecule. CD123 is the interleukin-3 receptor alpha chain expressed both at the level of leukemic stem cells and more differentiated leukemic blasts. Flotetuzumab application point at redirection of T lymphocytes to attack CD123-expressing cells. Flotetuzumab in a clinically applied dosage does not result in significant and prolonged suppression of normal hematopoiesis [16]. In non-human primates, no neutropenia or thrombocytopenia was observed [17].

While flotetuzumab has no FDA or EMA approval yet (as of January 2022), it is available in Japan and accessible in Europe through an expanded access program (NCT04678466). FDA has granted orphan drug designation to flotetuzumab for the treatment of AML. The only published clinical data available originate from a study in refractory AML [16] In this phase 1/2 study, 88 patients with refractory AML or intermediate/high-risk MDS were treated with several flotetuzumab dosages and schedules, some with addition of ruxolitinib and the anti-IL6 antibody tocilizumab. Patients with active infections were excluded. Flotetuzumab-related infections were not observed. FN was reported for 13.6% of 44 patients presented at the 2020 annual meeting of the American Society of Hematology (ASH) [18].

Risk of infection associated with flotetuzumab:

- No specific risks of infection in patients on flotetuzumab monotherapy.

Recommended diagnostic procedures:

- Standard of care in AML and neutropenic fever and/or infections (A-IIr).

Treatment recommendations:

- Standard of care in neutropenic fever and/or infections (A-IIr).

Recommendations for prophylaxis:

- No specific recommendation due to lack of data when given as monotherapy.

Recommendations on how to handle the drug in case of infection:

- No specific recommendation due to lack of data when given as monotherapy.

### IDH-1 and IDH-2 inhibitors (enasidenib, ivosidenib, olutasidenib)

Isocitrate dehydrogenases (IDH)-1 mutations are found in 6–10% of the patients with AML, IDH-2 mutations in 9–13%. Mutations in IDH-1 and -2 are almost always mutually exclusive and are present in a variety of other malignancies such as myelodysplastic syndrome, angioimmunoblastic T-cell lymphomas, gliomas, cholangiocarcinomas, chondrosarcomas and medulloblastomas [19]. Enasidenib (against IDH-2), ivosidenib and olutasidenib (both against IDH-1) are orally available small molecules inhibiting mutant IDH. For olutasidenib, no clinical study results are available as yet. Enasidenib was approved for relapsed and refractory IDH-2 positive AML treatment by the FDA in 2017; ivosidenib was FDA approved in 2019 for newly-diagnosed AML with a susceptible IDH-1 mutation in patients who are at least 75 years old or who have comorbidities that preclude the use of intensive induction chemotherapy. The EMA approval for these compounds was withdrawn by their specific companies in 2020.

No specific immunosuppressive effect of enasidenib or ivosidenib has been reported as yet. Ivosidenib in contrast to enasidenib is predominantly metabolized by CYP3A4, resulting in an increased exposure to ivosidenib when given in combination with strong inhibitors of CYP3A4 including triazole antifungals. The ivosidenib package insert indicates a warning for an increased risk of QTc interval prolongation in patients given this drug in combination with moderate or strong CYP3A4 inhibitors.

IDH inhibitors can cause a severe differentiation syndrome which may mimic an infection. The potential symptoms include fever, acute respiratory distress, pulmonary infiltrates, pleural or pericardial effusion, hyperleukocytosis or renal impairment up to multiorgan failure.

In a phase 1 study in 258 patients with relapsed or refractory AML patients treated with ivosidenib monotherapy [20], febrile neutropenia was noted in 25.2% and pneumonia (all grades) in 14.7%. No association with the study drug was observed. In 179 patients treated with the standard dose of ivosidenib 500 mg daily, febrile neutropenia occurred in 28.5% of patients. In a subgroup of 34 patients ineligible for standard chemotherapy due to age and/or comorbidities, who were treated with 500 mg ivosidenib daily, febrile neutropenia and pneumonia were documented in 3 patients each [21].

In a dose-finding phase 1 study, ivosidenib in combination with azacitidine was administered to 23 AML patients unfit for standard chemotherapy [22]. Febrile neutropenia was noted in 43.5% and lung infection (no further details reported) in 17.4% of patients.

The IDH-2 inhibitor enasidenib as monotherapy was studied in 345 patients IDH-2 mutated, relapsed or refractory AML [23]. No febrile neutropenia nor any type of infection was reported among treatment-related adverse events occurring in more than 5% of patients.

In another report on enasidenib monotherapy in 17 patients with IDH-2 mutant MDS [24], pneumonia was noted in 5 patients (29%) without further details described, and 3 patients (18%) has a differentiation syndrome.

In an open-label, single-arm trial, enasidenib was given in 39 “unfit” patients with newly diagnosed, IDH-2 mutant AML [25]. No treatment-related grade 3-4 adverse event was observed, however, pneumonia was reported for 7 (18%) patients, while a treatment relation was denied.

Ivosidenib (500 mg/day) or enasidenib (100 mg/day) were combined with intensive remission induction and consolidation chemotherapy in 151 patients with newly diagnosed, IDH-1 or -2 mutated AML in a phase 1 study [26]. Fever was reported in 26.7% of ivosidenib and 33.3% of enasidenib combination therapy recipients during induction and in 22.9% and 28.3% of patients during consolidation. Notably, clostridia infections were documented in 13.3% and 18.3% of patients during induction and in 5.7% and 10.9% of patients during consolidation therapy. While this has been a multicenter study, no discussion on a specific center effect or any other explanation for this unusual finding is provided. Ivosidenib/enasidenib monotherapy arms were not included, so that it remains unclear, if febrile events or clostridial infections were attributable to the IDH inhibitor.

Risk of infection associated with enasidenib or ivosidenib:

- No specific risks of infection in patients on enasidenib or ivosidenib monotherapy.

Recommended diagnostic procedures:

- Standard of care in AML and neutropenic fever and/or infections (A-IIr).

- In combination with more intensive antileukemic treatment, clostridia infections were reported from a single center.

Treatment recommendations:

- Standard of care in neutropenic fever and/or infections (A-IIr).

Recommendations for prophylaxis:

- No systemic antimicrobial prophylaxis when given as monotherapy (A-IIr).

Recommendations on how to handle the drug in case of infection:

- No specific recommendations

#### Gilteritinib

Gilteritinib is a highly selective oral FLT3 (Fms-Like Receptor Tyrosine Kinase 3) inhibitor with activity against both FLT3 mutation subtypes (Internal Tandem Duplication and Tyrosine Kinase Domain). It has a weak activity against KIT, suggesting a lower risk of myelosuppression when used for leukemia treatment. Gilteritinib also inhibits the tyrosine kinase AXL, resulting not only in a reduction of proliferation of AML cells, but also in immune suppression associated with AXL activity [27]. Gilteritinib is approved for the treatment of relapsed and refractory FLT3-mutated AML by FDA and EMA.

In an open-label phase 1/2 study, gilteritinib in a dose of 20–450 mg/day was given to 252 patients with relapsed/refractory AML irrespective of FLT3 mutation status [28]. Febrile neutropenia was documented in 40% of patients, with sepsis in 14%, pneumonia in 4%, and clostridial colitis in 2% of patients.

When randomized in a dosage of 120 mg/day versus salvage chemotherapy in a phase 3 study in 247 patients with FLT3-mutated, relapsed or refractory AML [29], febrile neutropenia was noted in 46.7% of patients given gilteritinib vs 36.7% in patients undergoing salvage chemotherapy (difference not significant), while treatment-related pneumonia was reported for 14.4% of patients treated with gilteritinib vs 17.3% of patients undergoing salvage chemotherapy.

Gilteritinib co-administered with strong CYP3A4 inhibitors (such as itraconazole, posaconazole or voriconazole) or inducers (such as rifampicin, rifamycin, rifapentine or rifaximin) may cause an increase or decrease of gilteritinib levels [30]. When one of the above-mentioned triazole antifungal is given concomitantly with gilteritinib, a dose reduction does not appear to be required, however, a close monitoring of potential QT interval prolongation is recommended. Whenever possible, the concomitant administration of gilteritinib and a strong CYP3A4 inducer should be avoided. Prescribing information should be noted (https://www.accessdata.fda.gov/drugsatfda_docs/label/2019/211349s001lbl.pdf).

Risk of infection associated with gilteritinib:

- While no specific impact on immune defense has been reported, infections reported in relapsed and/or refractory AML patients on gilteritinib are sepsis, febrile neutropenia and pneumonia.

Recommended diagnostic procedures:

- Standard of care in AML and neutropenic fever and/or infections (A-IIr).

Treatment recommendations:

- Standard of care in neutropenic fever and/or infections (A-IIr).

Recommendations for prophylaxis:

- No systemic antimicrobial prophylaxis when given as monotherapy (A-IIr).

Recommendations on how to handle the drug in case of infection:

- No specific recommendations.

Recommendations on how to handle the drug in case of concomitant administration of strong CYP3A4 inhibitors (e.g., itraconazole, posaconazole, voriconazole):

- Close monitoring for QT interval prolongation (A-I).

Recommendations on how to handle the drug if current medication includes a strong CYP3A4 inducer:

- Avoid combination.

#### Midostaurin

Midostaurin inhibits multiple tyrosine kinase receptors, including FLT3 and KIT kinases. It induces cell cycle arrest and apoptosis via the inhibition of FLT3 receptor signaling in AML cells with mutant FLT3 internal tandem duplications (ITD), tyrosine kinase domain (TKD) mutated receptors or overexpression of wild-type FLT3 receptors. In addition, midostaurin inhibits many other tyrosine kinase receptors such as platelet derived growth factor receptor (PDGFR), vascular endothelial growth factor receptor 2 (VEGFR2) or members of the serine/threonine kinases of the protein kinase C (PKC) family. Midostaurin in combination with chemotherapeutic agents (cytarabine, doxorubicin, idarubicin or daunorubicin) results in a synergistic inhibition of the growth of AML cell lines expressing FLT3-ITD. Apart from neutropenia, midostaurin has no impact on immune defense.

Midostaurin is approved for the treatment of adults with newly diagnosed flt3-mutated AML. Used upfront in combination with chemotherapy, midostaurin is continued as monotherapy for maintenance therapy after favorable response. Midostaurin is also approved for the treatment of aggressive systemic mastocytosis and mast cell leukemia. The prescription information for midostaurin contains a warning against combination of this drug with strong CYP3A4 inhibitors and more explicitly with strong CYP3A4 inducers.

Eleven phase 1 to 4 studies published between 2010 and 2020 were included in this review. Study populations consisted of patients with either newly diagnosed or relapsing/refractory AML. Midostaurin was used as monotherapy in two studies and in combination with standard chemotherapeutic regimens in nine. The median number of patients included was 69 (range: 11-717). The proportion of patients with febrile neutropenia ranged from 20% to 82% (median: 35%). Only four studies provided information on the frequency of infections [31–34] and four reported on the frequency of sepsis [34–37]. Pneumonia was the most frequent type of infection. It was reported in seven studies at a frequency ranging from 2% to 23% (median: 9%) [31, 35–40]. Other types of infections included upper respiratory tract infections (including sinusitis), cellulitis and bacteremias. The frequency of sepsis ranged from 4% to 18%. Bacterial infections tend to be frequent when midostaurin is used in combination with other cytotoxic drugs and much less so when used in monotherapy. Very few studies reported on the occurrence of fungal and viral infections which occurred at low frequencies (<10%) [36, 41]. This included one episode of pulmonary mycosis and two episodes of unspecified mycoses requiring antifungal therapy with an azole [34, 36] and seven episodes of herpes simplex infections [41].

Risk of infection associated with midostaurin:

- No specific impact on immune defense apart from neutropenia.

Recommended diagnostic procedures in case of fever:

- Standard of care for fever during cytarabine/anthracycline chemotherapy plus midostaurin therapy (A-IIr).

Treatment recommendations:

- Use of empirical antibiotic therapy during cytarabine/anthracycline chemotherapy plus midostaurin therapy in neutropenic fever and/or infections (A-IIr).

Recommendations for antimicrobial prophylaxis:

- Standard of care when midostaurin is given in combination with cytarabine/anthracycline chemotherapy (A-IIr).
- No need for antibacterial or antifungal prophylaxis when given as monotherapy (A-IIr).

Recommendations for concurrent use of CYP3A4 inducers or inhibitors:

- Warning for drug-drug interaction (see package insert): strong CYP3A4 inhibitors (clarithromycin, antifungal triazoles, cobicistat, antiviral protease, integrase, NS5A or polymerase inhibitors used for HIV and/or hepatitis C treatment) may increase exposure to midostaurin or its metabolites. Consider alternative therapies that do not strongly inhibit CYP3A4 or monitor for increased risk of adverse reactions.
- Avoid concomitant use of strong CYP3A4 inducers such as rifampin.

#### Quizartinib

Quizartinib is a potent and selective FLT3 tyrosine kinase inhibitor with significant activity in FLT3-ITD-mutant AML. The quality and duration of achievable response thus far seen with this agent as monotherapy is suboptimal. Quizartinib in combination with chemotherapy might result in improved outcome. Although the drug was granted FDA breakthrough designation in 2018, quizartinib has not been approved by FDA nor by EMA. To date, only the Ministry of Health, Labor and Welfare (MHLW) of Japan has approved quizartinib for the treatment of adult patients with relapsed/refractory *FTL3*-ITD-positive AML. When used to treat AML, the most common side effects with quizartinib were infections: 19% sepsis/septic shock, 12% pneumonia; grade 3 QT prolongation was seen in 3–4% of patients [42].

In a phase 1 study of quizartinib monotherapy administered daily (orally at escalating doses of 12–450 mg/day) to 76 patients with relapsed or refractory AML irrespective of FLT3-ITD status, only two cases of fever (3%) and one lung infection (1%, not otherwise specified) was noted [43]. Similar results were described in two other phase 1 studies. When quizartinib (40–60 mg/day) was given as maintenance therapy in 13 subjects with AML in remission following allogeneic cell transplantation, the most common grade 3/4 adverse events were hematologic (including 23% neutropenia and 15% lymphopenia) with only one patient (8%) developing ‘pneumonia’ [44]. When combined with ‘standard of care’ chemotherapy in 19 newly diagnosed AML patients, 47% of patients developed febrile neutropenia (all grade 3); the authors did not report specific data on infections. Both studies concluded that attention should be given to the co-administration with QT prolonging drugs [45]. In a large, open-label, multicenter phase 2 study, a total of 333 patients with relapsed/refractory AML received quizartinib (135 mg/day for men; 90 mg/day for women) monotherapy. Febrile neutropenia grade 3 and 4 developed in 37% and 4% of patients, respectively. Thirty-one patients (9%) had grade 3 pneumonia; 4% had grade 4 or 5 pneumonia. Different grades of sepsis ware noted in 25 patients (8%), ‘lung infection’ in 11 patients (3.3%) bacteremia in 10 patients (3%), cellulitis in 9 patients, fungal pneumonia in 9 patients, urinary tract infection (all grade 3) in 8 patients, and septic shock in 7 patients, respectively. Seven individuals (2.1%) developed Clostridium difficile colitis [46]. Additional phase 1 and 2 studies did not reveal any specific infectious risk related to quizartinib monotherapy in relapsed/refractory AML patients: febrile neutropenia and fever of unknown origin occurs in 21–43% of cases, pneumonia in 12–21% and sepsis in 4–11% [47, 48]. In the open-label phase 3 QuANTUM-R trial, 245 patients with relapsed/refractory FLT3-ITD AML received quizartinib 60 mg/day and 122 patients received chemotherapy. The most common non-hematological grade 3–5 adverse events were sepsis or septic shock (46 patients [19%] for quizartinib vs 18 [19%] for chemotherapy) and pneumonia (29 [12%] vs eight [9%]). The most frequent treatment-related serious adverse events were febrile neutropenia (18 patients [7%]), sepsis or septic shock (11 [5%]), and QT prolongation (5 [2%]) in the quizartinib group, and febrile neutropenia (5 [5%]), sepsis or septic shock (4 [4%]), pneumonia (2 [2%]) in the chemotherapy group. Of note, Clostridium difficile infection was seen in 11 quizartinib-treated patients vs none in the chemotherapy group. Seven patients in the quizartinib group developed fungal pneumonia (3%) versus five in the chemotherapy group (5%) [49].

Co-administration of quizartinib with drugs that prolong the QT/QTc interval and moderate or strong CYP3A inducers was prohibited in the QuANTUM-R trial. Avoidance of strong CYP3A inhibitors was recommended but not prohibited; however, if they were used, quizartinib dose adjustments were required. Weak or moderate CYP3A inhibitors, such as fluconazole, were allowed without dose reduction.

Risk of infection associated with quizartinib:

- No specific impact on immune defense apart from neutropenia. Febrile neutropenia/FUO and bacterial infections are frequent, mainly sepsis, urinary tract infection or upper respiratory infection. Fungal or viral infections appear to be very rare.

Recommended diagnostic procedures:

- Standard of care in AML and neutropenic fever and/or infections (A-IIr).

Treatment recommendations:

- Use of empirical antibiotic approach in neutropenic fever and/or infections (A-IIr).

Recommendations for antibiotic prophylaxis:

- Standard of care in AML, when given in combination (A-IIr).

Recommendations for combination with CYP3A4 inducing or inhibiting agents:

- See recommendations for gilteritinib.

#### Glasdegib

Glasdegib is a small molecule inhibitor of sonic hedgehog, which is a protein overexpressed in many types of cancer. The Hedgehog pathway inhibitor glasdegib has been shown to chemosensitize leukemic stem cells (LSCs). It inhibits the sonic hedgehog receptor smoothened (SMO), a key mediator of Hedgehog pathway signaling in LSCs. Glasdegib works synergistically with low-dose cytarabine (LDAC). No specific impact on immune defense apart from neutropenia has been identified as yet.

Glasdegib is approved, in combination with LDAC, for the treatment of newly diagnosed de novo or secondary AML (excluding acute promyelocytic leukemia) in adult patients who are not candidates for standard induction chemotherapy. Glasdegib is given continuously as long as the patient is deriving clinical benefit.

In a phase 1 study on glasdegib monotherapy in 28 patients with relapsed or refractory AML, neutropenia was documented in one patient [50].

In a randomized phase 2 study, glasdegib in combination with low-dose cytarabine was compared with low-dose cytarabine alone for first-line treatment of AML or high-risk MDS patients unsuitable for intensive chemotherapy [51]. Febrile neutropenia was reported for 35.7% vs 24.4% of patients, and pneumonia in 28.6% vs 24.4% of patients on combination vs low-dose cytarabine monotherapy.

When combined both with standard-dose cytarabine and daunorubicin (“7 + 3”), febrile neutropenia (mostly grade 3) was documented in 63.8% of patients, pyrexia in 49.3% and pneumonia and sepsis each in 10.1% of patients [52].

No specific risks of infection in patients on glasdegib monotherapy was reported. In clinical trials on glasdegib +LDAC, no signal of an increased risk of neutropenic fever/infections due to glasdegib was noted. Overall, febrile neutropenia, pneumonia and sepsis were reported in patients on combination glasdegib and intensive chemotherapy, with no potentially glasdegib-related infections.

Glasdegib is metabolized by CYP3A4/5, thus it is not recommended to use other drugs which inhibit CYP3A (such as erythromycin, clarithromycin, ciprofloxacin, posaconazole, voriconazole). A combination with strong CYP3A4 inducers such as rifampicin (see above) should be avoided [53].

Risk of infection associated with glasdegib:

- No specific impact on immune defense apart from neutropenia.

Recommended diagnostic procedures:

- Standard of care in AML and neutropenic fever and/or infections (A-IIr).

Treatment recommendations:

- Standard of care in neutropenic fever and/or infections (A-IIr).

Recommendations for prophylaxis:

- Standard of care in AML, when given in combination with chemotherapy (A-IIr).

Recommendations on how to handle the drug in case of infection:

- No specific recommendations.

Recommendations on combination with CYP3A4 inhibiting or inducing agents:

- Critical reconsideration of concomitant erythromycin, clarithromycin, ciprofloxacin, itraconazole, posaconazole, ketoconazole or voriconazole.
- Avoidance of combination with strong CYP3A4 inducers (see above).

#### Venetoclax

*For a previous ECIL review on venetoclax used for lymphoma treatment, see* [54].

Venetoclax is a BH(BCL2-homology)-3 mimetic that blocks the anti-apoptotic B-cell lymphoma-2 (BCL-2) protein, resulting in programmed cell death of leukemia cells. Overexpression of BCL-2 is contributing to lymphoid and myeloid malignancies. No specific impact on immune defense apart from neutropenia has been identified so far.

Venetoclax is approved in combination with a hypomethylating agents such as azacitidine or decitabine for patients with newly diagnosed AML (excluding acute promyelocytic leukemia) who are ineligible for intensive chemotherapy.

In a single arm phase 2 study on high-dose venetoclax (800 mg/day) monotherapy in 32 patients with relapsed or refractory AML unfit for intensive chemotherapy [55], febrile neutropenia (all grade 3-4) was reported for 31% and pneumonia for 25% of patients.

In a pivotal randomized placebo-controlled phase 3 trial, venetoclax (target dose of 400 mg/day) in combination with azacytidine (*n* = 283) was compared with azacitidine monotherapy (*n* = 144) in newly diagnosed AML patients ≥75 years of age unfit for standard chemotherapy [56]. Febrile neutropenia was reported for 30% vs 10% of patients (log-rank test *p* < 0.001), while pneumonia and sepsis were reported for 17% vs 22% and 6% vs 8% of patients on combination vs monotherapy, respectively. Infections of any grade occurred in 84% of the patients in the azacitidine–venetoclax group vs 67% of those in the azacytidine monotherapy group.

In a phase 1 study on venetoclax in a dose-escalation from 400 to 800 to 1200 mg/day, each in combination with azacitidine or decitabine, in elderly “unfit” patients with newly diagnosed AML [57], febrile neutropenia grade 3-4 was documented in up to 61% of patients, without an association with venetoclax dosage or one of the two hypomethylating agents. A low frequency of fungal infections (8% grade 3-4), despite exclusion of CYP3A inhibiting azole antifungals, which may be related to the prophylactic use of alternative antifungals such as echinocandins in 46% of patients.

A meta-analysis of eight reports on venetoclax in combination with hypomethylating agents in patients with AML or MDS [58] showed a febrile neutropenia rate of 47% (95% confidence interval 36–58%).

Venetoclax is metabolized by CYP3A4/5, specific attention must be paid when combined with other drugs which inhibit CYP3A (such as erythromycin, ciprofloxacin, triazoles). For the administration of posaconazole, as standard antifungal prophylaxis in AML patients undergoing myelosuppressive remission induction chemotherapy, an evidence-based recommendation for venetoclax dose reduction by 75% is available [59].

Risk of infection associated with venetoclax:

- No specific impact on immune defense apart from neutropenia.

Recommended diagnostic procedures:

- Standard of care in AML and neutropenic fever and/or infections (A-IIr).

Treatment recommendations:

- Standard of care in neutropenic fever and/or infections (A-IIr).

Recommendations for prophylaxis:

- Standard of care as for AML treatment with intensive chemotherapy (A-IIr).

Further recommendations for prophylaxis:

- Consider antibacterial and antifungal prophylaxis when hypomethylating agents are combined with venetoclax (A-IIr).

Recommendations on how to handle the drug in case of infection:

- Consider dose interruptions to allow for hematologic recovery in patients with a response (based on early bone marrow assessment, most importantly after the completion of cycle 1) (A-I).
- Promote appropriate interruptions in venetoclax between treatment cycles to augment hematologic recovery (A-I).
- In patients with good response but severe neutropenia, consider venetoclax dose reduction in subsequent courses (A-IIr).

- If dose reduction is ineffective or not advised, consider prophylactic granulocyte colony-stimulating factor during remission for subsequent courses (C-IIt).

Recommendations for concurrent use of venetoclax with antimicrobial agents:

- Ensure proper venetoclax dose adjustments when venetoclax is combined with antibacterial agents such as ciprofloxacin or macrolides (A-I).
- If posaconazole is given in combination with venetoclax, reduce venetoclax dose by 75% (A-I).
