# Correction: Infectious complications of targeted drugs and biotherapies in acute leukemia. Clinical practice guidelines by the European Conference on Infections in Leukemia (ECIL), a joint venture of the European Group for Blood and Marrow Transplantation (EBMT), the European Organization for Research and Treatment of Cancer (EORTC), the International Immunocompromised Host Society (ICHS) and the European Leukemia Net (ELN)

**DOI:** 10.1038/s41375-022-01570-9

**Published:** 2022-04-19

**Authors:** Georg Maschmeyer, Lars Bullinger, Carolina Garcia-Vidal, Raoul Herbrecht, Johan Maertens, Pierantonio Menna, Livio Pagano, Anne Thiebaut-Bertrand, Thierry Calandra

**Affiliations:** 1 Certified Cancer Center, Klinikum Ernst von Bergmann, Charlottenstrasse 72, D-14467 Potsdam, Germany; 2 Department of Hematology, Oncology and Tumor Immunology, Campus Virchow, Charité – Universitätsmedizin Berlin, German Cancer Consortium (DKTK), Partner Site Berlin, Berlin, Germany; 3 Infectious Diseases Department, Hospital Clinic-IDIBAPS, Villarroel 170, BCN 08036 Barcelona, Spain; 4 Department of Hematology, Institut de Cancérologie Strasbourg.Europe (ICANS) and Université de Strasbourg, Inserm, UMR-S1113/IRFAC, Strasbourg, France; 5 Department of Microbiology, Immunology, and Transplantation, KULeuven, Leuven and Department of Hematology, UZLEUVEN, Leuven, Belgium; 6 Department of Sciences and Technologies for Humans and the Environment, University Campus Bio-Medico of Rome, University Hospital Foundation Campus Bio-Medico of Rome, Rome, Italy; 7 Hematology Division, Dipartimento di Diagnostica per Immagini, Radioterapia Oncologica ed Ematologia, Fondazione Policlinico Universitario A. Gemelli – IRCCS and Università Cattolica del Sacro Cuore, Rome, Italy; 8 Department of Hematology, Grenoble University Hospital, Grenoble, France; 9 Infectious Diseases Service, Department of Medicine, Lausanne University Hospital, University of Lausanne, CH-1011 Lausanne, Switzerland

**Keywords:** Epidemiology, Acute myeloid leukaemia

Correction to: *Leukemia* 10.1038/s41375-022-01556-7, published online 02 April 2022

Due to an error in the typesetting process of this article, the names of the authors have been set incorrectly. We have corrected this in the original article.

We apologise for the error.

